# Hybrid cheetah particle swarm optimization based optimal hierarchical control of multiple microgrids

**DOI:** 10.1038/s41598-024-59287-x

**Published:** 2024-04-23

**Authors:** Mohamed Ahmed Ebrahim Mohamed, Ahmed Mohamed Mahmoud, Ebtisam Mostafa Mohamed Saied, Hossam Abdel Hadi

**Affiliations:** 1 Electrical Engineering Department, Faculty of Engineering at Shoubra, Benha University, Cairo, Egypt; 2 College of Technology in Cairo, MISR International Technological University, Cairo, 11813 Egypt

**Keywords:** Cheetah optimization, Hierarchical control strategies, Hybrid optimization systems, Microgrids, Particle swarm optimization, Sustainable energy systems, Energy science and technology, Engineering

## Abstract

The emergence of microgrids arises from the growing integration of Renewable Energy Resources (RES) and Energy Storage Systems (ESSs) into Distribution Networks (DNs). Effective integration, coordination, and control of Multiple Microgrids (MMGs) whereas navigating the complexities of energy transition within this context poses a significant challenge. The dynamic operation of MMGs is a challenge faced by the traditional distributed hierarchical control techniques. The application of Artificial Intelligence (AI) techniques is a promising way to improve the control and dynamic operation of MMGs in future smart DNs. In this paper, an innovative hybrid optimization technique that originates from Cheetah Optimization (CHO) and Particle Swarm Optimization (PSO) techniques is proposed, known as HYCHOPSO. Extensive benchmark testing validates HYCHOPSO’s superiority over CHO and PSO in terms of convergence performance. The objective for this hybridization stems from the complementary strengths of CHO and PSO. CHO demonstrates rapid convergence in local search spaces, while PSO excels in global exploration. By combining these techniques, the aim is to leverage their respective advantages and enhance the algorithm's overall performance in addressing complex optimization problems. The contribution of this paper offering a unique approach to addressing optimization challenges in microgrid systems. Through a comprehensive comparative study, HYCHOPSO is evaluated against various metaheuristic optimization approaches, demonstrating superior performance, particularly in optimizing the design parameters of Proportional-Integral (PI) controllers for hierarchical control systems within microgrids. This contribution expands the repertoire of available optimization methodologies and offers practical solutions to critical challenges in microgrid optimization, enhancing the efficiency, reliability, and sustainability of microgrid operations. HYCHOPSO achieves its optimal score within fewer than 50 iterations, unlike CHO, GWO, PSO, Hybrid-GWO-PSO, and SSIA-PSO, which stabilize after around 200 iterations. Across various benchmark functions, HYCHOPSO consistently demonstrates the lowest mean values, attains scores closer to the optimal values of the benchmark functions, underscoring its robust convergence capabilities.the proposed HYCHOPSO algorithm, paired with a PI controller for distributed hierarchical control, minimizes errors and enhances system reliability during dynamic MMG operations. Using HYCHOPSO framework, an accurate power sharing, voltage/frequency stability, seamless grid-to-island transition, and smooth resynchronization are achieved. This enhances the real application's reliability, flexibility, scalability and robustness.

## Introduction

Traditional utility has faced several challenges in recent decades. One of the most challenging issues represents in the vulnerability of traditional grid to single points of failure, such as equipment malfunctions, natural disasters and cyberattacks^1^, which can lead to widespread power outages. In addition, integrating large-scale Renewable Energy Sources (RESs) into the traditional grid poses technical challenges because of variable generation, intermittency and the need for substantial grid upgrades^2^. Additionally, the conventional utility is heavily reliant on fossil fuel power plants, which contribute to the emission of carbon dioxide and the degradation of the environment. Moreover, the traditional utility model involves long-distance transmission and distribution of electricity, resulting in significant transmission losses^3^. Microgrids (MGs) are localized energy systems that can operate islanded or interconnected with the main power grid. They comprise several Distributed Energy Resources (DERs) such as synchronous generator based and RESs such as Photovoltaic systems (PVs), Wind Turbine systems (WTs), Energy Storage systems (ESs), and backup generators, along with control and monitoring mechanisms. Microgrids provide reliable and sustainable electricity supply to specific areas, such as communities, campuses, military bases and industrial complexes. The concept of MGs has recently been introduced as a solution for centralized utility which has attracted the attentions of researchers^4,5^. Microgrid control strategies are essential for preserving the stability and dependability of MGs. The control strategies are required to regulate frequency levels and voltage, coordinate the operation of various components, and manage power flow. A review of recent developments in the control and optimization of MGs is elaborated, and the optimal operation of MGs is achieved by efficiently managing the production, storage, and consumption of electricity^6^. The authors ensure that energy resources are used in the most cost-effective and reliable manner, considering factors such as load demand, energy prices, and system constraints. The microgrid optimal operation with integrated power-to-gas technology is introduced^7^. Microgrid control strategies optimize the usage of RESs such as PVs and WTs, which are variable and intermittent, matching the generation with the load demand and grid conditions. Additionally, energy storage systems are managed to ensure efficient utilization of excess renewable energy and enable its availability during periods of low generation^8^. Hierarchical control of MGs refers to the management and coordination of multiple interconnected microgrids within a larger system and the establishment of control structures and techniques at different levels to ensure reliable and efficient operation of the interconnected microgrids. MGs can achieve improved efficiency, coordination, flexibility, and reliability by implementing hierarchical control strategies. It enables voltage and frequency regulation, system optimization, and effective management of power sharing, leading to optimal operation and integration of the interconnected MGs. The laboratory implementation of three-level hierarchical microgrid control is conducted and validated^9^. Artificial Intelligence (AI) approaches have been used in various aspects of hierarchical control in MGs, such as Particle Swarm Optimization (PSO)^10^, Evolutionary Algorithms (EA)^11^, grey wolf Optimization (GWO)^12^, Genetic Algorithms (GA)^13^, and reinforcement learning^14^ to optimize the operation and decision-making processes in hierarchical control. These approaches enable finding optimal solutions that consider multiple objectives, such as cost minimization, load balancing, and emission reduction while considering system constraints and uncertainties. As a result of using AI approaches in the hierarchical control of MGs, decision-making abilities are improved, system performance is increased, adaptive and intelligent control actions are made possible, and efficient resource management is promoted^15–17^. In the previous literature, the various primary control techniques for current and voltage regulation, secondary control for voltage and current error correction, power sharing in microgrids and microgrid clusters, and tertiary control for power and energy management with a primary focus on minimal power loss and operational cost in a Direct Current (DC) microgrid system are studied^18^. A microgrid inverter control strategy based on dynamic feedforward compensation is proposed and tested^19^. Moreover, a mathematical model for an Improved Phase-Locked Loop (IPLL) for a microgrid integrated with PV system control is developed^20^. However, conventional hierarchical control of MGs has certain limitations. One of these limitations is uncertainties and variability in RES generation, grid conditions, and load demand, rather than the scalability issue. Traditional hierarchical control techniques may struggle to handle many interconnected MGs. As the system grows in complexity and size, the traditional control structure becomes more challenging to manage. In addition, it might be unable to change in response to dynamic operational needs and system conditions. AI optimization techniques can be addressed to mitigate the limitations of traditional control structures. Therefore, PSO was conducted to review the cost optimization of MGs^21^. The optimal coordinate control of the DC microgrid based on the hybrid PSO-GWO algorithm is suggested and implemented^22^. The optimal energy management and battery sizing is applied for grid-connected MGs by using GWO algorithm^23^, whereas an Improved GWO (IGWO) algorithm is presented to shorten the time of the final result with the most precision^24^. Moreover, the improved algorithm is used for solving placement and sizing of electrical energy storage system in MGs. The IGWO is employed to dynamically optimize the rated power of Distributed Generation (DG) of the tertiary controller^25^. Salp Swarm Inspired Algorithm (SSIA) is used to improve the dynamic response and power quality of an islanded microgrid^26^. Droop control of DC microgrid is achieved by using a hybrid SSIA optimization with PSO^27^. The Liver Cancer Algorithm (LCA) is a newly developed bio-inspired optimization algorithm that models the growth and advancement of liver tumours. Employing an evolutionary search methodology, it imitates the takeover process of liver tumours within the organ. The LCA aims to optimize liver cancer-related processes by emulating tumour behaviour within the liver^28^. The efficiency of the LCA relies on the calibre of its parameters and design. In the absence of proper tuning or optimization, the algorithm's efficacy may be compromised, thereby affecting its applicability in real-world scenarios. The Parrot Optimizer (PO) is a highly effective metaheuristic algorithm modelled after the foraging, nesting, communication, and aversion to stranger’s behaviours observed in parrots. Engineered to address a wide range of optimization challenges, it extends its applicability to include medical contexts^29^. LCA and PO are commonly classified as global search algorithms. Unlike local search algorithms, which focus on specific regions of the solution space, global search algorithms aim to explore the entire solution space comprehensively. By doing so, they can identify the optimal solution, even when it is not readily apparent and may exist in various parts of the search space. This broad search capability makes them particularly effective for tackling complex optimization problems. The Slime Mould Algorithm (SMA) draws inspiration from the foraging behaviour of slime Molds, employing swarm intelligence principles to optimize solutions. Mimicking the oscillatory behaviour observed in slime moulds, SMA utilizes heuristics informed by experiences within specific regions (or patches) to decide when to move on, mirroring the natural behaviour of these organisms^30^. The Moth Search Algorithm derives its inspiration from the behaviour of moths drawn to light sources, employing the principle of phototaxis to guide its optimization process^31^. Similarly, the Hunger Games Search algorithm takes inspiration from the survival of the fittest concept depicted in the Hunger Games series, employing competitive selection processes to seek optimal solutions^32^. The Colony Predation Algorithm draws inspiration from the predatory dynamics within colonies, simulating interactions between predators and prey to refine solutions^33^. The Harris Hawks Optimization algorithm, inspired by the hunting patterns of Harris's hawks, relies on cooperative efforts and communication among agents to seek optimal solutions. As for the Rime Optimization Algorithm, while its inspiration may stem from rime ice formation^34^.

The salp swarm algorithm is used to improve the detection of Alzheimer's disease^35^. More specifically, the study aims to enhance the performance of the fuzzy K-nearest neighbours’ algorithm in Alzheimer's disease detection through optimization with the salp swarm algorithm. By employing advanced optimization methods in medical diagnostics, this investigation highlights the potential to enhance disease detection accuracy and efficiency, ultimately leading to improved patient outcomes. This algorithm is constrained by its vulnerability to local optima and reduced convergence accuracy during later iterations. An enhanced version of the Sine Cosine Algorithm (SCA) called the Hierarchical Multi-Leadership SCA (HMLSCA) is developed and tested to guide the optimization process along multiple paths^36^. The SCA might encounter challenges with low optimization searching accuracy, potentially hindering its efficiency in finding optimal solutions. This drawback could result in suboptimal outcomes, particularly in complex optimization scenarios^37^.

A modified particle swarm optimization algorithm tailored to address a batch-processing machine scheduling problem characterized by arbitrary release times and non-identical job sizes is introduced^38^. Novel machine learning methodologies are applied for fault diagnosis and optimization^39–41^. Machine learning models are frequently referred to as "black boxes," as they lack transparency regarding decision-making processes. This lack of transparency can impede comprehension of the rationale behind outcomes, thereby complicating the validation or accurate interpretation of results.

### The research gaps

The review of current literature on microgrid control methods and recent advancements in artificial intelligence (AI) optimization techniques has identified a gap in the application of bio-inspired optimization methods within hierarchical control systems for complex microgrid environments. While previous research extensively explored the use of AI algorithms, the integration of bio-inspired optimization techniques, especially those inspired by animal behaviours, remains largely unexplored in microgrid control systems.

The primary objectives of the proposed system are to ensure equitable power sharing and balance among multiple microgrids, maintain voltage and frequency stability by minimizing deviations, facilitate seamless transitions between grid-connected and islanded modes while minimizing the associated time and disruptions, and achieve smooth resynchronization of microgrids following islanding events.

The conducted research aims to bridge this identified gap in the literature by exploring how the integration of bio-inspired optimization techniques, demonstrated by the proposed hybrid cheetah optimization and particle swarm optimization system (HYCHOPSO), can significantly enhance the efficiency and adaptability of hierarchical control systems within microgrid operations. Specifically, the focus is on optimizing PI controller parameters to meet key performance targets, including ensuring equitable power sharing among multiple microgrids, maintaining voltage and frequency stability, enabling smooth transitions between grid-connected and islanded modes with minimal disruptions, and achieving seamless resynchronization of microgrids following islanding events.

The major contributions of this article can be summarized as follows:

- A new hybrid optimization system that merges the hunting strategy of CHO with the exploration property of PSO has been developed.
- A comparative study is performed to demonstrate the effectiveness of the proposed HYCHOPSO with various types of metaheuristic optimization approaches, including single as well as hybrid algorithms.
- The developed HYCHOPSO has been proposed to solve one of the most popular microgrid technical problems presented in the optimal design of PI controller parameters of hierarchical control systems.

The remainder of this paper is organized as follows: In Section "Proposed hybrid optimization system", the proposed hybrid optimization framework, encompassing mathematical models for PSO and CHO is developed. This section also provides the pseudocode for the novel HYCHOPSO, conducts rigorous testing against five other optimization systems, showcases the superior performance of the new hybrid system over the alternatives, and presents an in-depth analysis of the results, including the convergence curves derived from various benchmark testing functions. Section "Multiple microgrids control strategies" illustrates multiple microgrid control strategies, including conventional and hierarchical control techniques. The application of AI, especially the newly proposed optimization system for the hierarchical control of multiple microgrids, is demonstrated in section "Application of HYCHOPSO on hierarchical controlfor multiple microgrid". Section "Simulation result and discussion" presents the results and discussion. Finally, section "Conclusion" presents the conclusion.

## Proposed hybrid optimization system

A hybrid optimization system refers to a combination of different optimization algorithms or techniques such as p-metaheuristic, s-metaheuristic, machine learning, and mathematical programming that are integrated to improve algorithm efficiency, reduce search time, provide better quality solutions, improve effectiveness, provide accuracy, and solve complex optimization problems^42–50^. Exploration and exploitation are typically the two different phases of the process in metaheuristic optimization. These phases are essential for the search procedure to successfully navigate the solution space and locate superior solutions^51^. During the exploration phase, the metaheuristic algorithm focuses on exploring a global search of the solution space, agents travel in the search space, update the agent position, and update the best agent. The metaheuristic algorithm then moves on to the exploitation phase, where it focuses on refining and exploiting the promising solutions identified during exploration. This phase performs solution evaluation, selection, mutation, crossover, and target update. In the proposed system, the exploration phase is represented by PSO, and the exploitation phase is represented by CHO.

### Particle swarm optimization

PSO is a high-quality, human-based, global-based, and intelligence-based algorithm that finds a solution to the optimization problem^52^. One of the bio-inspired algorithms, PSO, is straightforward in its search for the best solution in the problem area. It differs from conventional optimization algorithms in that it doesn’t rely on the gradient or any differential forms of the goal; instead, it only needs the objective function. The mathematical equation of the PSO can be presented as the following Eqs. (1)–(3):

1 $$\begin{array}{c}\overline{{X }_{n+1}^{i}}=\overline{{X }_{n}^{i}+\overline{{V }_{n+1}^{i}}}\end{array}$$

2 $$\begin{array}{c}\overline{{V }_{n+1}^{i}}=w\overline{{V }_{n}^{i}+c1r1(\overline{{p }_{n}^{i}-\overline{{X }_{n}^{i})}}}+c2r2 \left(\overline{{p }_{n}^{g}}-\overline{{X }_{n}^{i}}\right)\end{array}$$

3 $$\begin{array}{c}W={W}_{max}-\left(\left({W}_{max}-{W}_{min}\right)*\left(\frac{1}{Maxiter}\right)\right)\end{array}$$

where r1, r2 are random number; i: particle index n: number of iterations [0,1]; ${V}_{n}^{i}$:V velocity of particle i at iteration n; ${X}_{n}^{i}$ :X position of particle i at iteration n; c1 and c2: coefficients of optimization parameter which are usually between [0 2] each velocity update.; ${p}_{n}^{i}$: local best position.; ${p}_{n}^{g}$ : global best position;

W: PSO Inertia Constant.;${{\text{W}}}_{{\text{max}}, }{{\text{W}}}_{{\text{min}}}\mathrm{ are the maximum and minimum inertia damping}.$

The advantages of PSO are the capability to change the position of particles in a multidimensional search space. This unique advantage paves the way for employing PSO in tackling various engineering challenges such as voltage and frequency control on interconnected power systems, maximum energy harvesting of both solar as well as wind energy conversion systems, energy management of RESs and stabilizing of inverted pendulum^53–69^ On the other hand, the most important disadvantage of PSO is that the regulation speed and direction of the particle are not exact. This method may not perform well in non-coordinate systems^70^.

### Cheetah optimization

This optimization technique is motivated by the hunting strategy of cheetah^71^. Cheetahs generally use three strategies for hunting prey, which can be summarized as follow:

- Searching, Cheetahs need to search, including scanning or active search, in their territories (search space) or the surrounding area to find their prey.
- Sitting and waiting, after the prey is detected, but the situation is not proper, cheetahs may sit and wait for the prey to come nearer or for the position to be better.
- Attacking which has two essential steps:
- Rushing: When the cheetah decides to attack, they rush toward the prey with maximum speed.
- Capturing: The cheetah uses speed and flexibility to capture the prey by approaching the prey.

The mathematical equation of CHO can be presented by the following Eqs. (4)–(6):

In searching strategy:

4 $$\begin{array}{c}{X}_{i,j}^{t+1}={X}_{i,j}^{t}+{{\widehat{r}}_{i,j}}^{-1}{\propto }_{i,j}^{t}\end{array}$$

In sit and wait strategy:

5 $$\begin{array}{c}{X}_{i,j}^{t+1}={X}_{i,j}^{t}\end{array}$$

In the attack strategy:

6 $$ \begin{array}{*{20}c} {X_{i,j}^{t + 1} = X_{B,j}^{t} + \overset{\lower0.5em\hbox{$\smash{\scriptscriptstyle\smile}$}}{r}_{i,j} B_{i,j}^{t} } \\ \end{array} $$

where ${X}_{i,j}^{t+1},{X}_{i,j}^{t}$ are the next and current position of cheetah (i = 1, 2,…,n), n is the population size in arrangement (j = 1, 2,…,D), and D is the dimension; ${\widehat{r}}_{i,j}^{-1},{\propto }_{i,j}^{t} \,are\, the\, randanization \,parameterand\, step\, length \,for \,cheetah\, i.$

The step length higher than zero, in most cases, can set at 0.001*t/T where t is the current hunting time and T is the maximum length of hunting time. Cheetah optimization has the advantages of fast convergence, adaptive behaviour, simplicity and ease of implementation, and robustness. However, it has a limitation of lack of extensive research, limited exploration capability^71^. Therefore, recent research has moved toward hybridizing with another technique to obtain its advantages while avoiding its limitations.

### Proposed hybrid system

Over the past few decades, many metaheuristic optimization techniques had been applied to deal with different engineering problems^72–101^.

In the realm of optimization strategies, a “hybrid optimization system” refers to the integration of various algorithms and techniques, including p-metaheuristics, s-metaheuristics, machine learning, and mathematical programming. This integration aims to enhance algorithmic efficiency, reduce search time, improve solution quality, increase effectiveness, ensure accuracy, and effectively address complex optimization problems.

The conventional metaheuristic optimization paradigm comprises two essential phases: exploration and exploitation, both crucial for efficiently navigating the solution space and discovering superior solutions. The exploration phase involves the metaheuristic algorithm conducting a comprehensive global search across the solution space. Agents iteratively traverse the search space, updating their positions, and refining the best agent. Subsequently, the algorithm transitions seamlessly into the exploitation phase, where it focuses on refining and exploiting promising solutions identified during the exploration phase. This phase encompasses critical activities such as solution evaluation, selection, mutation, crossover, and target updates.

In our proposed system, we assign the exploration phase to Particle Swarm Optimization (PSO), where the algorithm actively explores the extensive global solution space. Conversely, the exploitation phase is represented by Cuckoo Search-based Hybrid Optimization (CHO), which emphasizes refining and exploiting solutions identified during the preceding exploration phase.

### Pseudocode of the new optimization technique HYCHOPSO

The randomization parameters are illustrated in Eqs. (7)–(10). In Eq. (10), c2 is modified from the existing one in the original Cheetah version. In Eq. (12), the modification of the existing one in the original PSO optimization to update the leader’s follower step. The modified mathematical model for calculating the position of the leader is presented as follows:

7 $$\begin{array}{c}{r}_{Hat}=randn\end{array}$$

8 $$\begin{array}{c}A=rand\left(\right)\end{array}$$

9 $$\begin{array}{c}B=rand\left(\right)\end{array}$$

10 $$\begin{array}{c}C2=B-(\frac{{\text{A}}-{\text{B}}}{Maximumiteration})\end{array}$$

11 $$\begin{array}{c}\overline{{V }_{n+1}^{i}}=w\overline{{V }_{n}^{i}+c2*r1(\overline{{X }_{best}^{i}-\overline{{X }_{n}^{i})}}}\end{array}$$

12 $$\begin{array}{c}\overline{{V }_{n+1}^{i}}=w\overline{{V }_{n}^{i}+c2*r1(\overline{{X }_{b}^{i}-\overline{{X }_{n}^{i})}}}\end{array}$$

13 $$\begin{array}{c}{X}_{i,j}^{t+1}={X}_{i,j}^{t}+W*{r}_{Hat}*\overline{{V }_{n+1}^{i}} \end{array}$$

where ${r}_{Hat}$, A, B, and C2 are randomization numbers; ${X}_{best}^{i},\overline{{X }_{n}^{i}}, {X}_{b}^{i}$ are the prey’s position, Cheetah’s current position, and cheetah’s best position.

**Figure:**
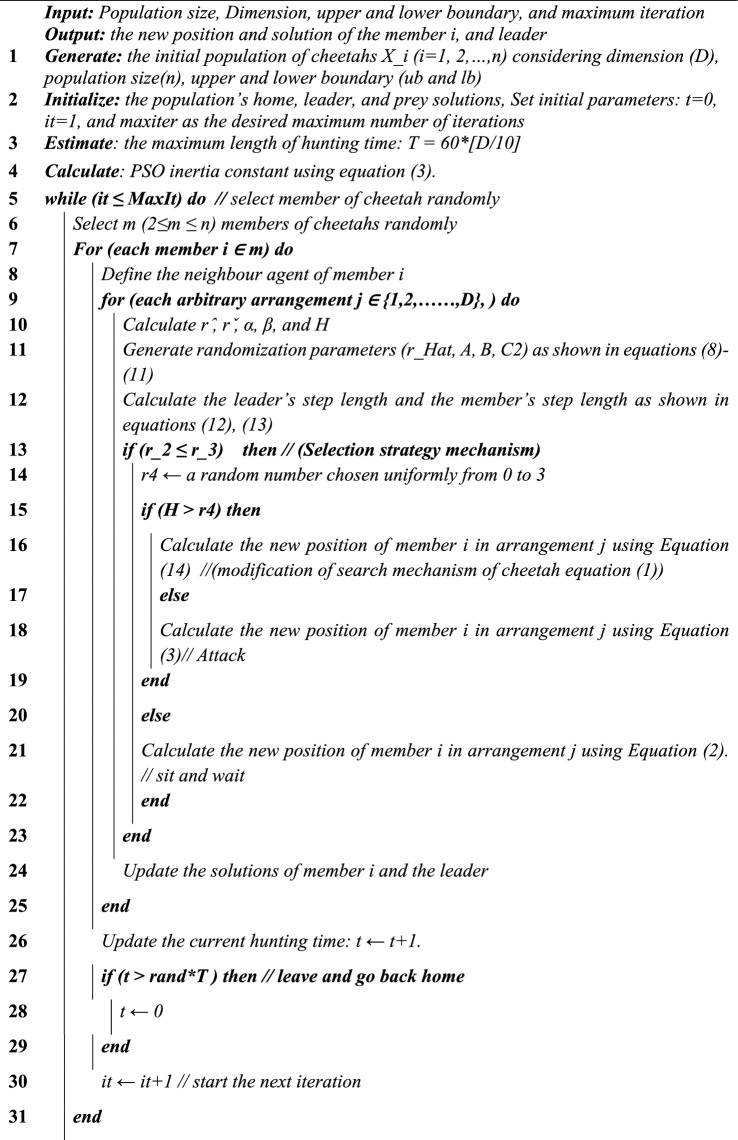
Algorithm: the new proposed optimization technique HYCHOPSO

### Comparative study between the proposed HYCHOPSO with alternative metaheuristics on benchmark functions

**Figure 1:**
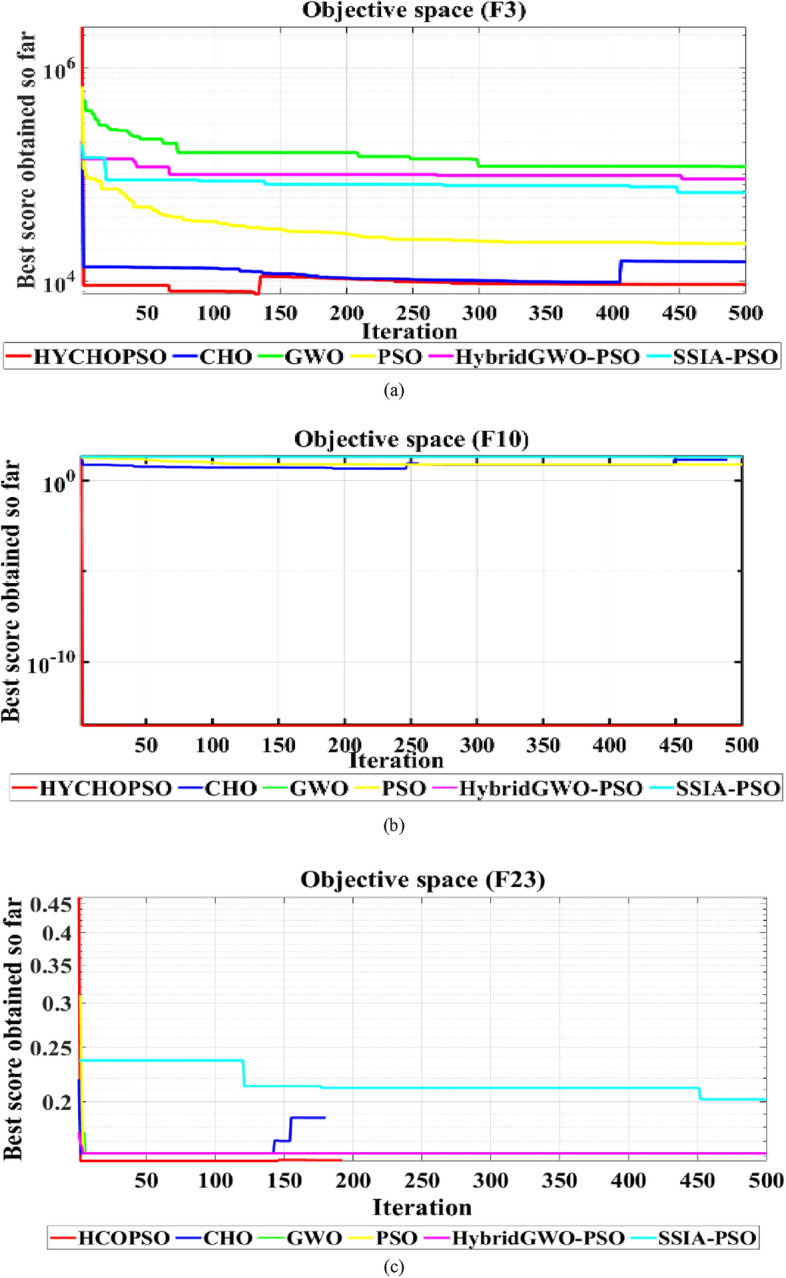
Convergence curves for testing benchmark functions (**a**) F3, (**b**) F10, (**c**) f23.

The hybrid HYCHOPSO algorithm is applied to twenty-three benchmark functions in this segment. The benchmark problems consist of three groups: unimodal, multimodal, and fixed-dimension multimodal functions. Convergence curves are the most common results in the single-objective optimization literature. Moreover, the mean and standard deviation values can be used as Performance Indicators (PIs) for the enhanced behaviour of HYCHOPSO compared with other alternative metaheuristics. The obtained results and convergence performance indicate that HYCHOPSO is more reliable. Figure 1 shows sample results for the convergence characteristics of HYCHOPSO, CHO, GWO, SSIA-PSO, PSO, and GWO-PSO during application to F3, F10, and F23.

The analysis of different optimization systems with the novel HYCHOPSO is implemented under the same conditions to make a fair comparison, which depends on the following strategies:

- The optimization problem is clearly defined for different categories of unimodal, multimodal, and fixed-dimension multimodal benchmark functions.
- Each optimization system is implemented separately, following their specific algorithmic details and parameter settings. i.e., setting the random seeds, the same initial conditions, stopping rules, and termination criteria.
- The best score obtained at systematic intervals (e.g., after each iteration or generation) is recorded during the optimization process, and this data is stored for each optimization system.
- Finally, the convergence curves are plotted for each optimization system using the stored data.

The maximum number of iterations was adjusted to 500 and the number of search agents was 6. The capability test for HYCHPSO, CHO, GWO, PSO, hybrid GWO-PSO, and SSIA-PSO has 6 runs per function. HYPCHOPSO illustrates its superiority in most benchmark functions over alternative optimization methods. The results obtained from the convergence performance showed that the proposed HYCHOPSO is more reliable. Table 1 reports the average and standard deviation of the statistical results over 6 runs. Table 1 shows that HYCHOPSO has the best results in F1, F2, F3, F4, F5, F6, F7, F10, F13, F22, and F23. Based on previous results, HYCHOPSO has the best performance because it combines the advantages of both CHO and PSO.

**Table 1:** Comparative statistical analysis for various meta-heuristics during application to benchmark functions.

Testing function		HYCHOPSO	CHO	GWO	PSO	Hybrid-GWO-PSO	SSIA-PSO
F1	Standard	**1.77E−07**	5.89E+03	9.03E+04	5.69E+02	6.18E+04	4.97E+04
	Average	**2.01E−07**	1.28E+04	9.01E+04	2.50E+03	6.18E+04	5.80E+04
F2	Standard	**1.32E**+**01**	27.01627	1.16E+09	23.79372	4.01E+03	3.11E+06
	Average	**2.84E**+**02**	1.82E+12	3.41E+09	4.93E+07	4.01E+03	3.11E+06
F3	Standard	**9.34E**+**03**	1.52E+04	1.52E+05	2.27E+04	8.23E+04	6.79E+04
	Average	**9.47E**+**03**	1.21E+04	1.58E+05	2.77E+04	1.05E+05	7.66E+04
F4	Standard	**7.46E**+**00**	72.96569	96.62468	29.37893	98.39876	78.86015
	Average	**3.8527**	74.1908	96.4314	34.0721	98.4008	81.7905
F5	Standard	**4.41E**+**03**	1.46E+06	2.34E+08	2.27E+05	3.94E+08	1.68E+08
	Average	**1.77E**+**03**	3.44E+07	2.34E+08	4.01E+06	3.94E+08	1.76E+08
F6	Standard	**7.83E−07**	1.20E**−**04	8.12E+04	185.8541	5.05E+04	4.82E+04
	Average	**8.64E−07**	0.7463	8.10E+04	1.94E+03	5.05E+04	5.45E+04
F7	Standard	**1.48E−01**	2.90E+00	1.29E+02	1.75E**−**01	2.03E+02	4.85E+01
	Average	**1.30E−01**	9.13E**−**01	1.29E+02	1.67E+00	2.03E+02	6.43E+01
F8	Standard	− 7.48E+03	− **9.17E**+**03**	− 3.59E+03	− 7.10E+03	− 4.51E+03	− 3542.93
	Average	− 1.05E+04	− **1.01E**+**04**	− 3.50E+03	− 6.54E+03	− 4.47E+03	− 3.41E+03
F9	Standard	1.06E+02	**7.68E**+**01**	4.73E+02	1.08E+02	4.62E+02	3.82E+02
	Average	7.45E+02	**6.50E**+**01**	4.72E+02	1.49E+02	4.78E+02	3.95E+02
F10	Standard	**2.93E−14**	14.2E+00	2.00E+01	7.73E+00	2.01E+01	2.02E+01
	Average	**2.93E−14**	7.12E+00	1.99E+01	7.55E+00	2.01E+01	2.02E+01
F11	Standard	5.32E**−**01	**2.04E−01**	7.71E+02	5.61E+00	5.52E+02	4.37E+02
	Average	5.20E+00	**1.61E−01**	7.69E+02	2.01E+01	5.52E+02	4.52E+02
F12	Standard	2.25E**−**03	**2.11E−04**	1.03E+09	6.01E+00	5.89E+08	2.69E+08
	Average	2.10E**−**03	**3.92E−04**	1.03E+09	3.65E+06	5.90E+08	2.97E+08
F13	Standard	**2.86E+00**	6.27E+00	1.70E+09	2.76E+02	1.53E+09	6.76E+08
	Average	**3.89E−01**	7.28E+00	1.70E+09	4.86E+06	1.53E+09	7.15E+08
F14	Standard	**9.98E−01**	**9.98E−01**	1.93E+01	**9.98E−01**	2.02E+00	1.11E+01
	Average	**9.98E−01**	**9.98E−01**	4.00E+01	2.28E+00	3.52E+00	1.44E+01
F15	Standard	2.59E**−**03	9.66E**−**04	2.26E**−**02	**6.92E−04**	2.13E**−**03	1.35E**−**02
	Average	1.90E**−**03	8.58E**−**04	4.19E**−**02	**5.80E−04**	7.00E**−**03	1.88E**−**02
F16	Standard	**− 1.03E+00**	**− 1.03E+00**	**− 1.03E+00**	**− 1.03E+00**	**− **1.02E+00	**− **7.8E−01
	Average	**− 1.03E+00**	**− 1.03E+00**	**− **8.97E−01	**− 1.03E+00**	**− **1.01E+00	1.34E+00
F17	Standard	**3.98E−01**	**3.98E−01**	1.02E+00	**3.98E−01**	**3.98E−01**	5.67E**−**01
	Average	**3.98E−01**	**3.98E−01**	1.15E+00	4.35E**−**01	4.37E**−**01	7.10E**−**01
F18	Standard	**3.00E+00**	**3.00E+00**	3.91E+00	9.18E+01	3.21E+00	1.01E+01
	Average	**3.00E+00**	**3.00E+00**	5.87E+00	9.33E+01	4.33E+00	2.79E+01
F19	Standard	**7.48E+00**	**7.48E+00**	7.55E+00	7.55E+01	7.55E+00	2.43E**−**04
	Average	1.57E**−**07	1.90E**−**07	**1.51E−07**	**1.51E−07**	**1.51E−07**	4.87E**−**07
F20	Standard	2.73E**−**07	6.46E**−**07	**5.53E−08**	1.44E**−**06	5.88E**−**07	3.21E**−**05
	Average	2.10E**−**05	1.88E**−**07	**1.24E−07**	5.79E**−**05	7.89E**−**07	2.87E**−**04
F21	Standard	1.57E**−**01	1.62E**−**01	**1.52E−01**	0.161823	1.62E**−**01	2.04E**−**01
	Average	**1.57E−01**	1.58E**−**01	1.62E**−**01	1.63E**−**01	1.62E**−**01	2.18E**−**01
F22	Standard	**1.53E−01**	1.73E**−**01	1.62E**−**01	1.62E**−**01	1.73E**−**01	1.98E**−**01
	Average	**1.53E−01**	1.73E**−**01	1.63E**−**01	1.62E**−**01	1.73E**−**01	2.05E**−**01
F23	Standard	**1.57E−01**	1.82E**−**01	1.62E**−**01	1.73E**−**01	1.62E**−**01	1.99E**−**01
	Average	**1.57E−01**	1.82E**−**01	1.63E**−**01	0.1734	1.62E**−**01	2.12E**−**01

Significant values given in bold.

### Computational cost analysis of the HYCHOPSO algorithm

In this section, we will discuss both the time and space complexity aspects associated with HYCHOPSO. The analysis of time complexity will focus on evaluating the computational efficiency of HYCHOPSO in terms of its runtime performance. This examination aims to provide insights into the algorithm's efficiency in handling various optimization tasks within acceptable time frames. Additionally, we will investigate the space complexity, considering the memory requirements of the algorithm during execution. This comprehensive analysis is intended to offer a transparent understanding of HYCHOPSO's computational characteristics, allowing readers to assess its practical feasibility and performance compared to existing algorithms. We will ensure that this section is presented in a clear and organized manner, providing the necessary information for a thorough evaluation of the computational aspects of HYCHOPSO.

The results obtained from Fig. 1a reveal that for benchmark function F3, the HYCHOPSO algorithm achieves the fastest convergence speed, reaching its optimal score before 50 iterations, while CHO, GWO, and PSO show slower convergence, stabilizing around 105 after approximately 200 iterations. The GWO-PSO and SSIA-PSO algorithms exhibit the slowest convergence speeds, with SSIA-PSO failing to stabilize within the displayed iterations. In Fig. 1b for Benchmark Function F10, the graph shows that HYCHOPSO, CHC, GWO, PSO, GWO-PSO, and SSIA-PSO converge rapidly to the optimal solution, maintaining their best scores within the initial 50 iterations throughout the 500 iterations. This indicates similar convergence speeds, enabling them to escape local minima and find global minima effectively. Turning to Fig. 1c for Benchmark Function F23, HYCHOPSO emerges as the top performer, converging quickly to a score above 0.4 within the first 50 iterations, showcasing its efficiency in finding near-optimal solutions. Conversely, GWO-PSO and SSIA-PSO exhibit poor performance, displaying minimal improvement and remaining near the bottom throughout 500 iterations, indicating challenges in optimizing effectively and being stuck in suboptimal solutions. The CHO, GWO, and PSO algorithms show moderate performance, with CHO displaying step-like progression, GWO slightly outperforming PSO by the end of iterations, and both exhibiting steady convergence compared to HYCHOPSO, GWO-PSO, and SSIA-PSO. In conclusion, the obtained results and convergence performance show that HYCHOPSO is more reliable.

The results presented in Table 1 provide a detailed analysis of the performance metrics for the HYCHOPSO algorithm compared to alternative algorithms across various benchmark functions (F1–F23). The values reported include both standard deviation and average (mean) for each testing function. Lower mean scores in functions F1, F2, F3, F4, F5, F6, F7, F10, F13, F22, and F23 indicate superior algorithm performance, with HYCHOPSO consistently exhibiting the lowest mean values compared to other algorithms. This signifies that, on average, HYCHOPSO achieves scores closer to the optimal values of the benchmark functions, indicating its robust convergence capabilities. Additionally, lower standard deviation values in most benchmark functions suggest more consistent and predictable results, reinforcing HYCHOPSO's stability and reliability across diverse optimization scenarios. Overall, the combination of low mean values and standard deviation values in Table 1 indicates that HYCHOPSO consistently outperforms alternative algorithms in terms of convergence speed and reliability across a wide range of benchmark functions. In summation, the observed convergence patterns in graphical representations substantiate the quantitative metrics presented in Table 1, collectively affirming that HYCHOPSO exhibits superior convergence speed and reliability compared to alternative algorithms across the specified benchmark functions.

## Multiple microgrids control strategies

### Conventional control

Voltage stability, frequency regulation, load frequency control and error minimization, power sharing, optimal power flow, and energy management are the key control and operational features of the traditional control architecture. Voltage stability is crucial for maintaining steady voltage levels within acceptable limits, achieved through mechanisms such as voltage regulators and reactive power compensation devices. Frequency regulation ensures the system frequency remains at its nominal value, typically 50 Hz or 60 Hz, through Automatic Generation Control (AGC) and governor control systems. LFC, a subset of frequency regulation, focuses on adjusting generation to match load demand in real-time, essential for system stability. Error minimization involves reducing deviations between desired setpoints and actual parameters, optimizing performance using control strategies like PID controllers and Model Predictive Control (MPC). Power sharing ensures equitable distribution of power among generators or Distributed Energy Resources (DERs) within the microgrid, facilitated by control algorithms such as droop control and consensus-based control. OPF addresses the optimal operating conditions of the power system, optimizing generation dispatch while satisfying constraints like voltage and power flow limits. Energy management encompasses planning, scheduling, and control of energy resources to meet demand efficiently, utilizing real-time data and advanced control algorithms within Energy Management Systems (EMS). These key control and operational features synergize to ensure the reliable, efficient, and sustainable operation of microgrids, especially in integrating renewable energy sources and accommodating dynamic changes in generation and demand. By effectively managing voltage, frequency, power flow, and energy resources, microgrid operators can optimize system performance and enhance grid resilience. Reference^92^ introduced a comparison to summarize the control and operational features of conventional control architecture. In the conventional control, maintaining the voltage, and frequency within limits are implemented by the Automatic Voltage Regulation (AVR), and turbine governor. However, these techniques are not suitable for penetration of RES such as PVs and ESs. Therefore, research tends to focus on alternative control techniques to mitigate the problems that occur with penetration RES on microgrid.

### Hierarchical control strategy of microgrid

The hierarchical control architecture comprises multiple layers, each serving distinct functions to ensure the stable and efficient operation of microgrids. The hierarchical control’s primary layer is used to make the system stable and damped by emulating the physical behaviour of the system which can be realized by adding a virtual impedance control loop through droop control. The primary hierarchical layer uses local controllers to achieve complex control. Therefore, this layer has a real-time response or is very fast. An enhanced master to slave control is also proposed for precise load sharing through parallel standalone AC microgrids^93^. One of the primary layer’s local controller possible challenges is the coordination control of the master-to-slave controllers itself.

In contrast, the secondary layer of hierarchical control focuses on compensating for and managing voltage and frequency deviations induced at the primary layer. This layer plays a crucial role in ensuring system stability by addressing fluctuations in voltage and frequency^100^. Moreover, it facilitates synchronization for seamless connection and disconnection from the main grid, enhancing operational flexibility and effectiveness.

At the apex of the hierarchical control architecture is the tertiary control layer, responsible for overseeing energy management and optimizing power flow between the microgrid and the main grid. This layer orchestrates the efficient utilization of energy resources and facilitates optimal power exchange between the microgrid and external sources. By coordinating energy flows and optimizing power dispatch, the tertiary control layer maximizes the overall efficiency and reliability of microgrid operation, contributing to enhanced grid resilience and sustainability.

### Phase locked loop (PLL) for PV inverter control

The ability of a PV system to successfully synchronize its output with the grid ensures effective power transmission and adherence to grid standards is achieved with a PLL control. PLL is a popular control mechanism used for grid synchronization, frequency tracking, phase control, and voltage regulation^100^. The voltage and the frequency of the utility signals is an input for the frequency detector which measures the frequency of the signal, then the phase of the grid signal compared with the output phase of the PV system by phase detector. The frequency variance is converted into a corresponding voltage signal by the Frequency Voltage Converter (FVC). The phase difference between the utility signal and the output of the PV system is converted into a frequency deviation signal by the Phase-to-Frequency Converter (PFC). The Low Pass Filter (LPF) removes high frequency noise while keeping the desirable low frequency components when filtering the output signals of FVC and PFC. The Voltage Controlled Oscillator (VCO) generates an oscillating signal related to the frequency controlled by LPF. The grid synchronization unit collects signals from VCO, LPS and sends these signals to inverter control which minimizes the deviation between the grid signal and the output of the PV array.

### Current regulator with feedforward control for PV inverter control

A current regulator with feedforward compensation can be employed in a microgrid to manage the output current of an inverter linked to a renewable energy source such as solar or wind and deliver the electricity to the grid. The input of PI controller is the error signals which is the subtraction of the measured direct or quadrature current from the reference values as shown in Eqs. (14) and (15). Then this error signal used as an input for PI controller for generating control signal Pid, and Piq which is shown in Eqs. (15) and (16).

14 $$\begin{array}{c}{E}_{id}={id}_{ref}-{id}_{meaured}\end{array}$$

15 $$\begin{array}{c}{E}_{iq}={iq}_{ref}-{iq}_{meaured}\end{array}$$

16 $$\begin{array}{c}Pid={K}_{p}*{E}_{id}+{K}_{i}*\int {E}_{id}*dt\end{array}$$

17 $$\begin{array}{c}Piq={K}_{p}*{E}_{iq}+{K}_{i}*\int \begin{array}{c}{E}_{iq}*dt \\ \end{array}\end{array}$$

where Eid, Eiq are the error signals in direct current and quadrature current; Pid, Piq are the control signal of pi controller for direct current and quadrature current error signals.

The feedforward compensation signals are Vd_measured and Vq_measured. The output of the feedforward compensation is shown in Eqs. (18) and (19). The output from the current regulator with feedforward compensation is shown in Eqs. (20) and (21).

18 $$\begin{array}{c}{Vd}_{feedforward}={Vd}_{measure}+{id}_{ref}*Rtot\\ \quad +{iq}_{ref}*Ltot\end{array}$$

19 $$\begin{array}{c}{Vq}_{feedforward}={Vq}_{measure}+{iq}_{ref}*Rtot\\ \quad  -{id}_{ref}*Ltot\end{array}$$

20 $$\begin{array}{c}{Vd}_{conv}={Vd}_{measure}+id*Rtot-iq*Ltot\\ +\frac{did}{dt}*\frac{Ltot}{Wbase}\end{array}$$

21 $$\begin{array}{c}{Vq}_{conv}={Vd}_{measure}+id*Ltot-iq*Rtot\\ +\frac{diq}{dt}*\frac{Ltot}{Wbase}\end{array}$$

Vdfeedforward, Vqfeedforward are the output of feedforward compensation for direct current signal and quadrature current signal; Vdconv, Vqconv are the output of the feedforward for direct and quadrature signals; Rtot, Ltot,wbase are total resistance, total inductance and base angular frequency.

### Droop control with PLL control technique

Droop control is a common control method for microgrid systems. It enables power sharing and voltage/frequency regulation among DERs. In a droop control, each DER adapted its power output based on local measurements and reference values. Equations (22), and (23) show the droop control for frequency and voltage which are related to active and reactive power.

22 $$\begin{array}{c}{P}_{out}={K}_{pf}*\left({F}_{PLL}-{F}_{ref}\right)+{K}_{if}*\int {(F}_{PLL}-{F}_{ref})dt\end{array}$$

23 $$\begin{array}{c}{Q}_{out}={K}_{pV}*\left({V}_{out}-{V}_{ref}\right)+{K}_{iV}*\int \left({V}_{out}-{V}_{ref}\right)dt\end{array}$$

The rate of change of frequency (RoCoF) and voltage (RoCoV) are also taken into consideration in the control system for ensuring stability as shown in Eqs. (24), and (25).

24 $$\begin{array}{c}{P}_{out}={P}_{out}+{K}_{pdf}*\left({D}_{Fref}-{R}_{o}{C}_{o}F\right)\\ \quad +{K}_{idf}*\int \left({D}_{fref}-{R}_{o}{C}_{o}F\right)dt\end{array}$$

25 $$\begin{array}{c}{Q}_{out}={Q}_{out}+{K}_{pdf}*\left({V}_{Fref}-{R}_{o}{C}_{o}V\right)\\ \quad  +{K}_{idf}*\int \left({D}_{Vref}-{R}_{o}{C}_{o}V\right)dt\end{array}$$

where Pout, and Qout are the output active and reactive power; Kpf, and Kif are the control parameter for pi control within active power for maintain stable frequency with varying power; Kpv, and Kiv are the control parameter for pi control within reactive power for maintain stable voltage with varying power; RoCoF, RoCo V are the rates of change of frequency and voltage. Kpdf, Kidf, Kpdv, Kidv, Dfref, Dvref are the drop control parameters.

The precise values of the controllers’ gains (Kpdf, Kidf, Kpdv, Kidv, Dfref, Dvref) can provide system stability based on the system requirement. For ensuring the synchronization between the microgrid and utility, a PLL control technique is used based on the calculation of theta from Eq. (26).

26 $$\begin{array}{c}{\theta }_{out}={\theta }_{out}+{K}_{Pppl}*\left({F}_{pll}-{F}_{ref}\right)\\ +{K}_{ipll}*\int \left({F}_{pll}-{F}_{ref}\right) \end{array}$$

where θ_out_: Output phase angle of the PLL controller; K_Ppll_: Proportional gain of the PLL controller; KIpll: Integral gain of the PLL controller; F_pll_: Output frequency of the PLL; F_ref_: Reference frequency.

The output frequency can be calculated from Eq. (27).

27 $$\begin{array}{c}{F}_{out}={F}_{PLL}+{F}_{ref}\end{array}$$

where F_out_: Output frequency of the system; F_PLL_: Output frequency of the PLL; F_ref_: Reference frequency.

## Application of HYCHOPSO on hierarchical controlfor multiple microgrid

**Figure 2:**
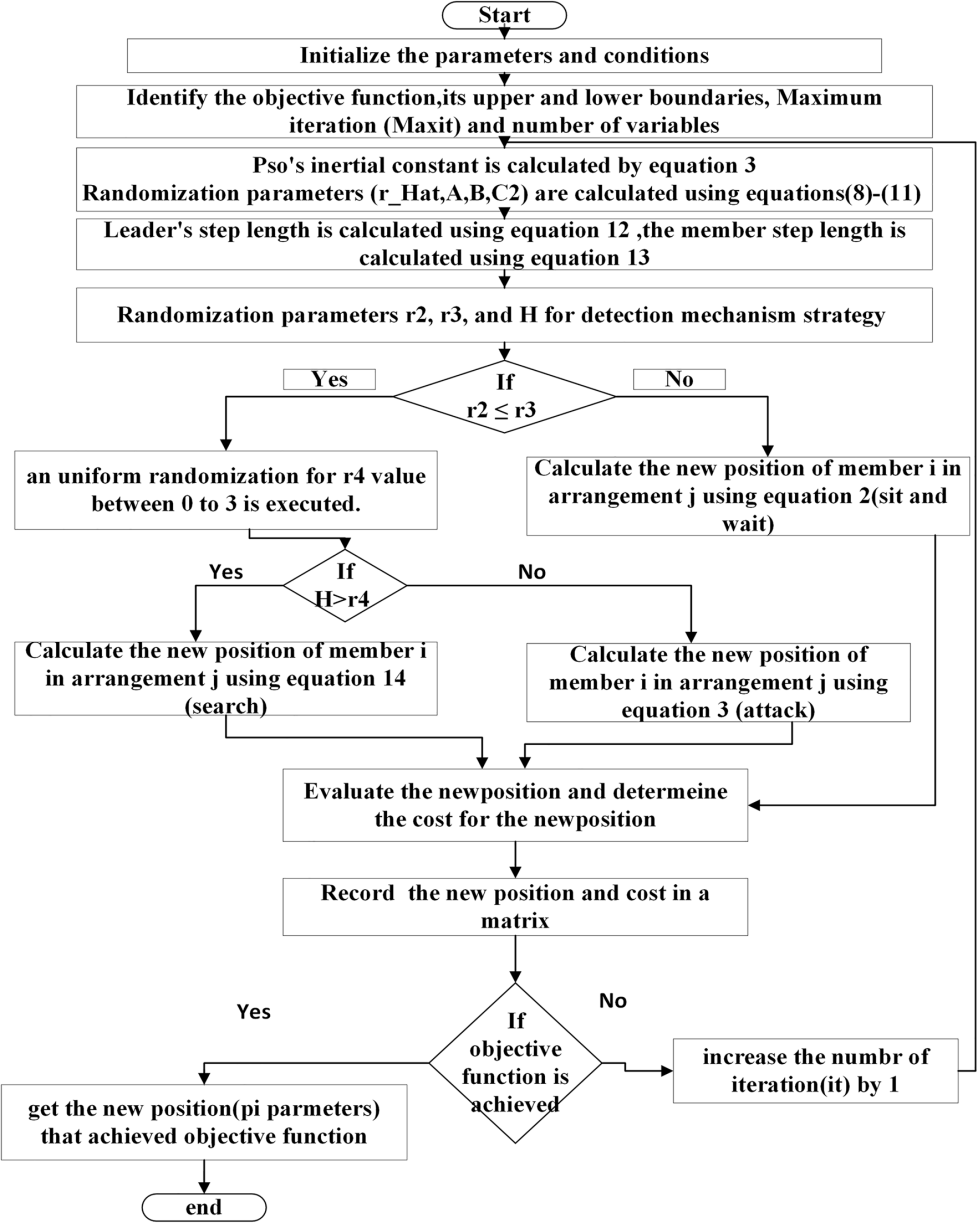
The flowchart of application HYCHOPSO for PI control parameter tuning used with Hierarchical control of multiple microgrid.

**Figure 3:**
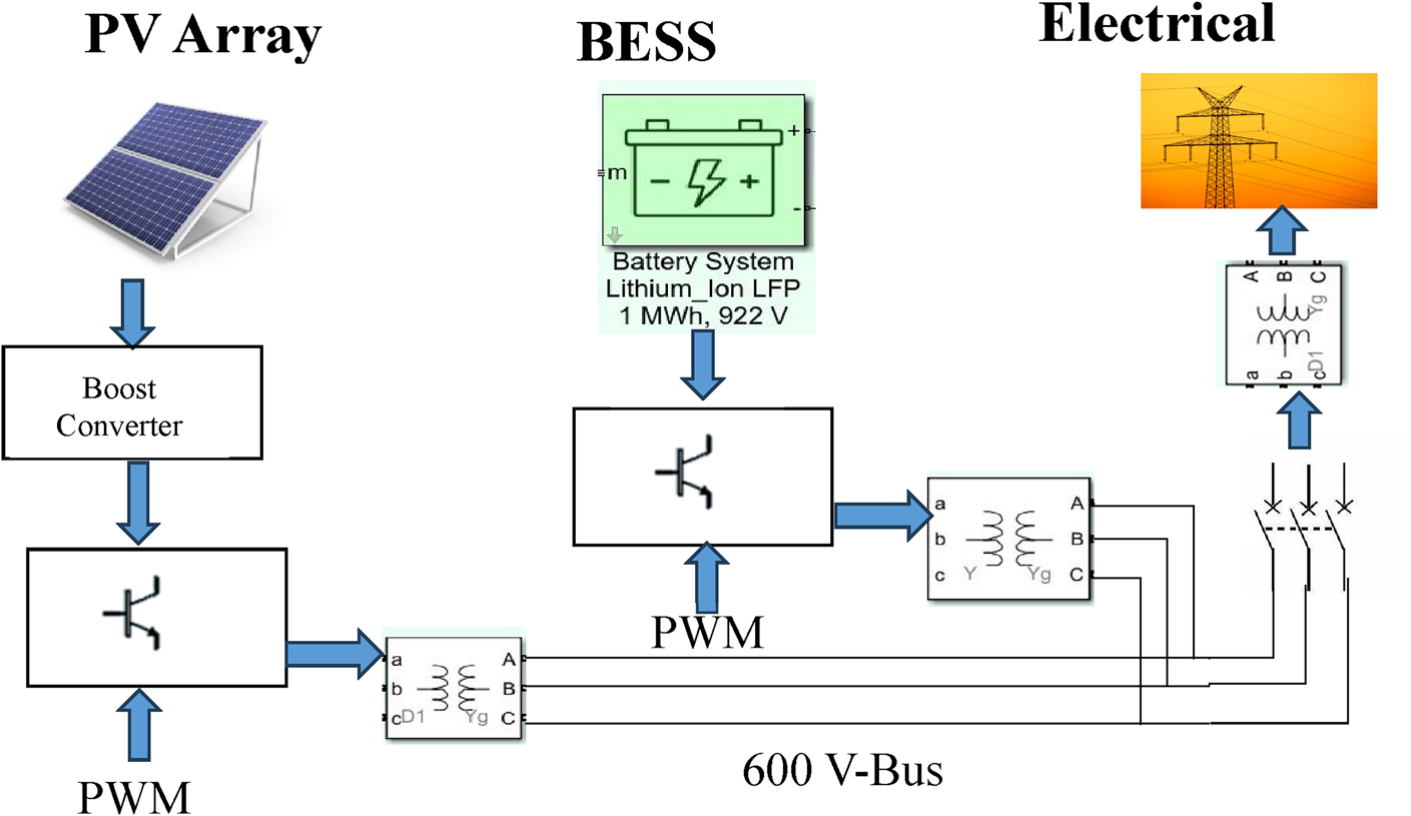
Configuration of simulation model with PV panel, BESS, and utility.

The proposed HYCHOPSO technique has demonstrated its effectiveness as an optimization tool, so it will be utilized to determine the best control parameters that achieve minimum error. Figure 2 illustrates the flowchart for the application steps of the proposed HYCHOPSO for tuning the PI controller parameters for the hierarchical control of multiple microgrids. The simulation model consists of PV plants, Battery Energy Storage System (BESS), and distribution system as shown in Fig. 3. Minimization of the controller’s error is the objective function of the optimization technique. The Integral of Absolute Error (IAE), Integral of Square Error (ISE), integral of time absolute error (ITAE), and Integral of Time Square Error (ITSE) are the four types of error benchmark objective functions^98^. The application of ITAE is the most appropriate for comparing the performance of PI controller with different optimization system^27^. The parameters of the test system are summarized in Table 2^102^. The HYCHOPSO for primary and secondary control levels on microgrid is coded using MATLAB/SIMULINK environment (Release: 2021a). The HYCHPSO well-tuned controllers are installed with multiple microgrids for effective hierarchical control for controlling the frequency and voltage, current regulation and ensuring power-sharing between multi-sources as well as.

**Table 2:** Test System Parameters [102].

Parameters	Value
PV system	
PV nominal DC power	1 MW
PV nominal Voltage	1000 V
Inverter nominal frequency	50 Hz
Transformer 1 nominal primary voltage	600 V
Transformer T1 nominal Secondary voltage	480 V
PV filter	L = 0.34 H, R = 0.00034 Ω, C = 0.01152 F
PV DC filter	L = 1*10^–3^ H, R = 5*10^–3^ Ω, C = 200*10^–6^ F
BESS system	
BESS nominal DC power	1 MW
BESS nominal voltage	922 V
BESS voltage limits	V_max_ = 1050 V, V_min_ = 750 V
Transformer T2 nominal primary voltage	600 V
Transformer T2 nominal secondary voltage	442 V
BESS filter	L = 0.026H, R = 0.026*10^–2^ Ω, C = 0.00976 F
BESS initial state of charge (SoC)	70%

## Simulation result and discussion

**Figure 4:**
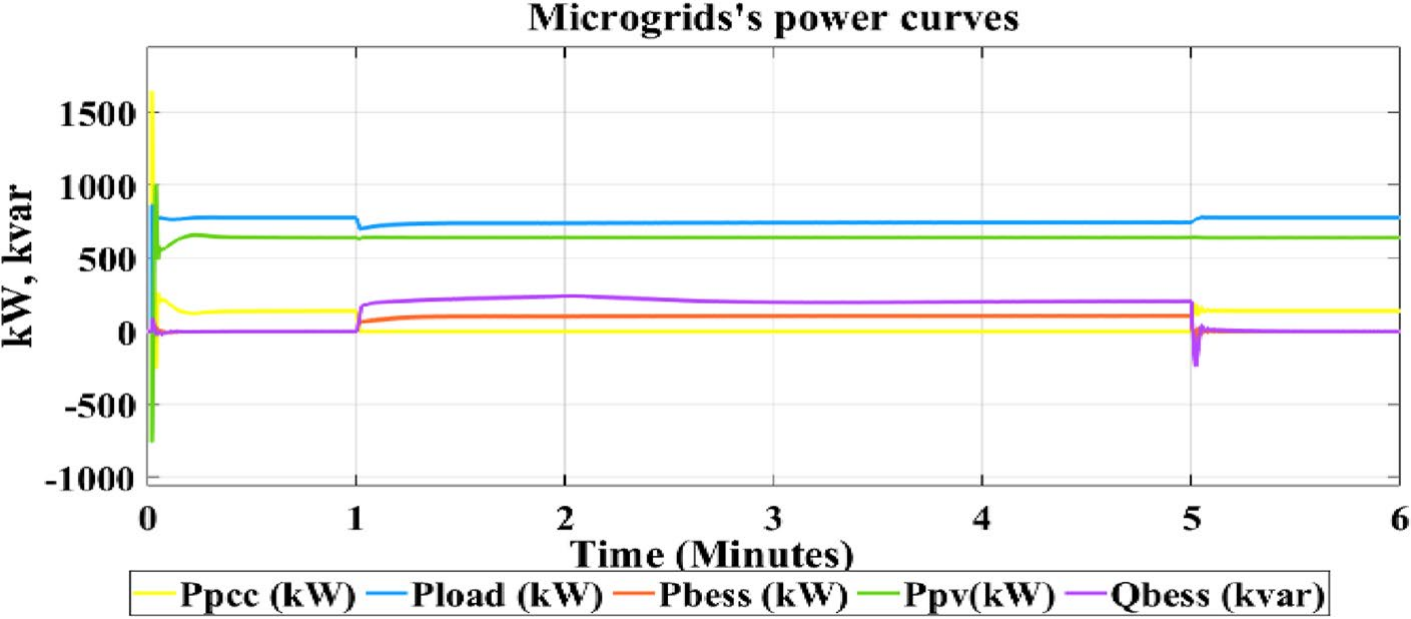
The active load power, active PV power, Active BESS power, Active power at PCC, and reactive BESS power.

**Figure 5:**
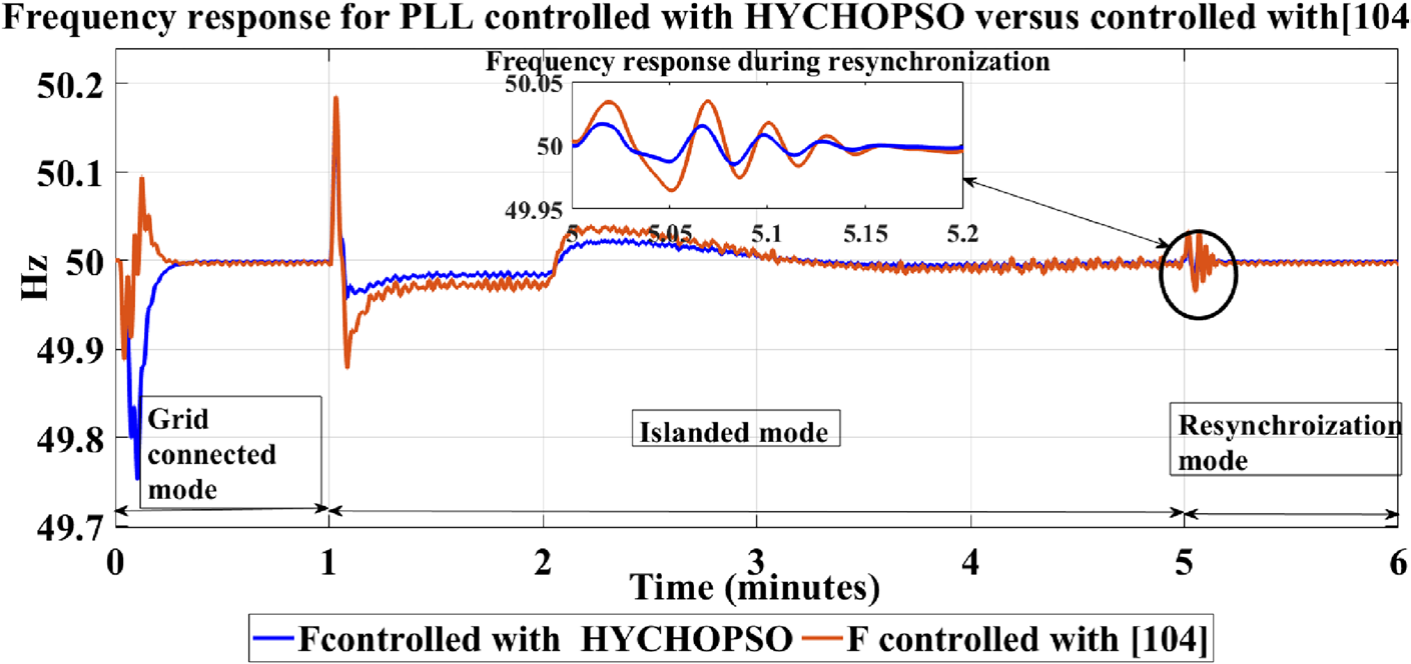
Comparing frequency signal for PLL with HYCHOPSO versus with tunning pi parameters [95].

Microgrids are operated in grid connected mode under 650 W/m^2^ fixed irradiation for photovoltaic array which is connected to BESS and 120 kV grid equivalent. Microgrids are operated in grid connected mode for 1 s then converted to islanded mode for 4 s and then connected again to grid through a resynchronization unit. The microgrid's response to different operating scenarios, including grid-following and grid-forming modes, is investigated. Figure 4 illustrates a detailed curve analysis on key parameters such as PV active power, BESS active and reactive power, active power at the Point of Common Coupling (PCC) and load active power. Figure 5 shows the output frequency of PLL with optimizing PI control parameters by using new proposed technique (HYCHOPSO), and with tuned PI control parameters technique^102^.

The observed behaviour from curves in Fig. 4 during load changes and system mode transitions provides valuable insights into the microgrid's stability using HYCHPSO-based control strategies. The response of active BESS curve illustrates that when the microgrids operate in a grid connected mode the BESS didn’t share with the PV active power to cover the load active power. But when the microgrids transition occurs, and the microgrids operated in islanded mode, the BESS shares with PV plant active power. This behaviour aligns with load variations and highlights the BESS's ability to provide additional power during peak demand. The BESS reactive power injected after 1 min when microgrids operate at islanded mode to ensure improving voltage stability and indicate the system's response to grid voltage variations and its ability to provide reactive power.

From Fig. 5, the frequency response curve tuned using the HYCHOPSO algorithm depicts an initial low-frequency fluctuation of around 49.75 Hz. This is followed by a gradual increase, ultimately reaching 50 Hz with a lower level of ripple compared to the reference-tuned curve. The curve's response demonstrates the capability of the HYCHOPSO technique to achieve frequency stabilization through dynamic adjustments of PI parameters. The curve associated with the PLL tuned using the traditional technique exhibits initial fluctuations, with the frequency oscillating around 50.095 Hz^102^. Over time, the fluctuations are damped, eventually stabilizing at 50 Hz with minimal ripple. The technique's effectiveness is evident in achieving stable frequency despite the initial fluctuations. So, the system controlled with HYCHOPSO make seamless transition from grid connected to islanded mode, and smooth resynchronization to the grid.

**Figure 6:**
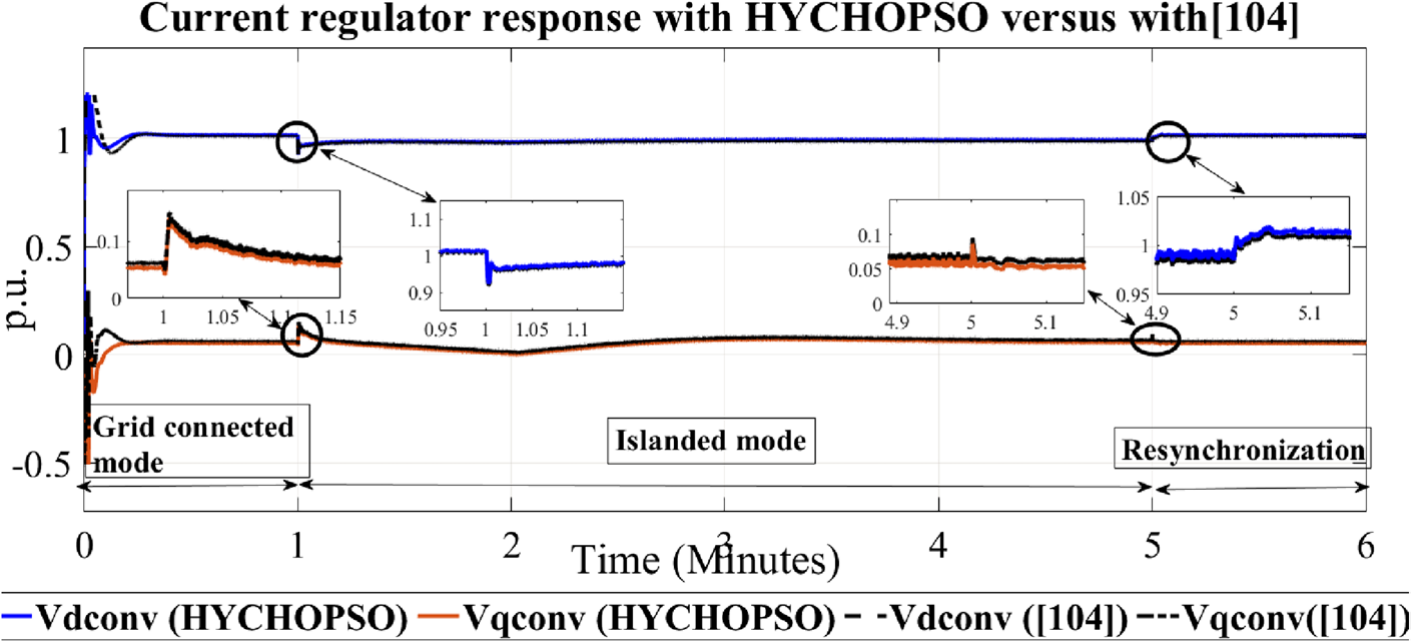
The output of current regulator controlled with HYCHOPSO versus the output controlled [102].

**Figure 7:**
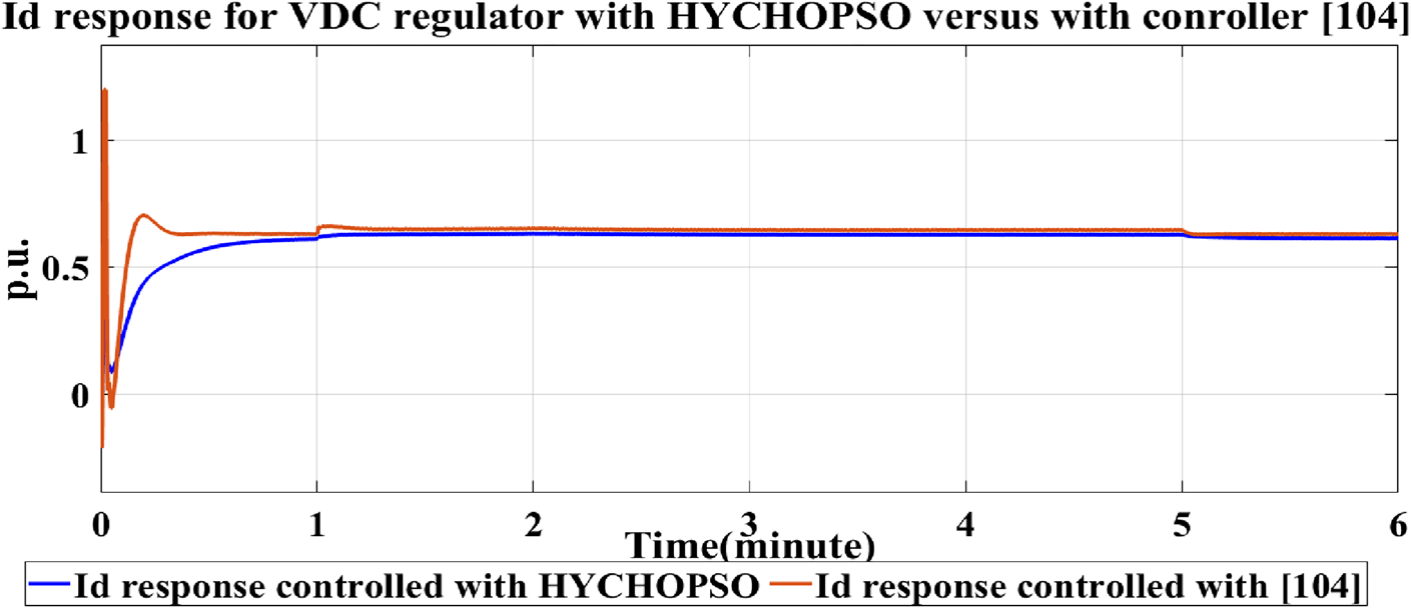
the output id signal from PVVDC regulator tuned with pi control optimized by HYCHOPSO.

The output signal from current regulator Vd, Vq by using HYCHOPSO for minimizing the error signal as described in mathematical Eqs. (14)–(21). The output voltage of the current regulator with feedforward regulation controlled by pi controller tuned with HYCHOPSO make the output voltage nearly constant during the period between islanded from utility till reconnection again more than these curves tuned with the traditional technique^102^. This occurred by reducing the error signals in id, and iq and producing control signals pid, and piq as shown in Fig. 6. Figure 7 shows the output of the VDC regulator id controlled with pi controller turned with HYCHOPSO versus the traditional technique^102^. The generation reference voltage is based on the output signals from the current regulator, and the output signal id results from VDC voltage regulator.

The analysis for curves in Fig. 6 illustrates that the output curve and testing methodologies showcase the differences between the HYCHOPSO and the traditional technique tuning techniques^102^. These differences reflect that each technique is employed to minimize error signals and achieve stable current regulation.

**Figure 8:**
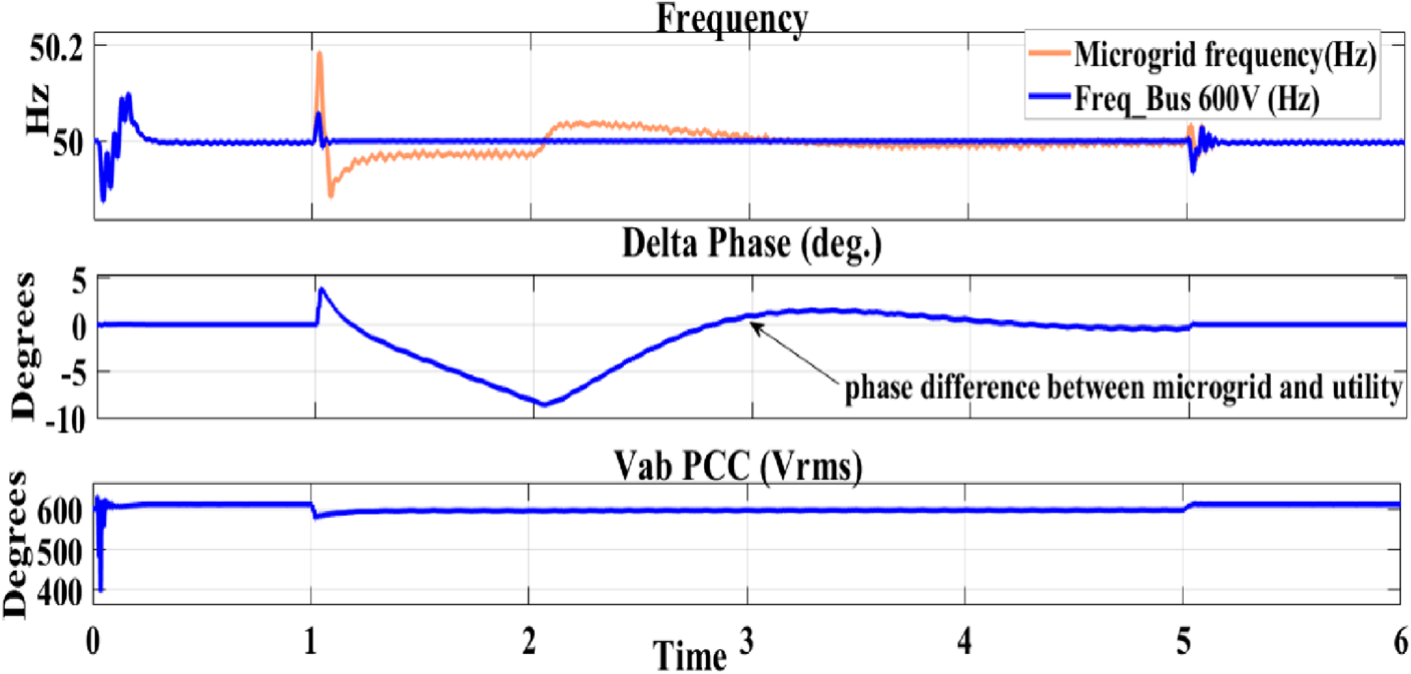
The output of droop controlled tuned with HYCHOPSO.

The id output signal curve of the PV VDC regulator tuned using HYCHOPSO demonstrates remarkable performance. The transient fluctuations are minimized swiftly, leading to a stable and accurate id output signal around the reference value. This indicates that the novel technique has the capability of achieving precise and rapid regulation. While the curve outlined using the traditional technique eventually stabilizes, the transient behaviour suggests a comparatively slower response^102^. This may lead to extended regulation settling times and potentially less accurate regulation during dynamic condition. The comparative analysis underscores the impact of the tuning technique on the PV VDC regulator's performance. The HYCHOPSO-tuned curve showcases a more rapid and accurate regulation process compared to the traditional technique tuned curve^102^. Figure 8 illustrates the output of the droop control technique controlled by HYCHOPSO. The droop control operates at a slower time scale and is responsible for achieving higher-level objectives such as voltage and frequency regulation, and it is used for confirming resynchronization.

The analysis of frequency curves shown in Fig. 8 illustrates the microgrid frequency during both grid-following and grid-forming modes. Figure 8 shows how droop control maintains frequency stability and tackles any deviations during transitions of the microgrid. The phase difference curve depicts the angular displacement between the grid and microgrid voltages. The phase angle remains at zero during grid-connected conditions and deviates during islanded microgrid operation. The curve illustrates that the droop control maintains voltage within acceptable limits during various modes while highlighting any variations during transitions. The curve analysis provides insights into how droop control affects. The very short Total Demand Distortion (TDD) was calculated according to IEEE519-2014 standardization and its value is 1.1% which is acceptable for 600 V-bus^103^.

## Conclusion

This study introduces a robust hybrid optimization algorithm namely HYCHOPSO that combines CHO and PSO. Its performance is evaluated on twenty-three benchmark problems and compared to seven other metaheuristic algorithms. The results demonstrate its effectiveness in PI controller tuning and hierarchical control for paralleled DGs. The presented study reveals valuable insights into microgrid stability using HYCHOPSO-based control strategies. It is observed that BESS efficiently shares power with the PV system during islanded mode, enhancing power availability during peak demand. Furthermore, the HYCHOPSO-tuned current regulator demonstrates superior performance in maintaining voltage stability compared to the technique, by minimizing error signals and producing stable current regulation. Additionally, droop control plays a crucial role in maintaining frequency stability during mode transitions and voltage limits during various operational modes. These findings highlight the effectiveness of HYCHOPSO in optimizing microgrid control, ensuring stable and reliable operation. The research outcomes present compelling evidence supporting the utilization of the HYCHOPSO algorithm for microgrid control, substantiated by quantitative data and insightful analyses. HYCHOPSO demonstrates its effectiveness in facilitating efficient power sharing between the photovoltaic (PV) system and the Battery Energy Storage System (BESS). The resynchronization unit ensures a seamless transition, stabilizing the active load power at 780 kW. Notably, the active power at the Point of Common Coupling (PCC) for Load increases to 184 kW after 5.055 min, highlighting the algorithm's ability to respond adeptly to mode transitions. The frequency response analysis underscores HYCHOPSO's precision, with the frequency stabilizing at 50 Hz with minimal ripple. Comparative analysis with a reference tuning method underscores HYCHOPSO's superior performance in mode transitions and stable frequency control. Examination of the PV VDC regulator accentuates HYCHOPSO's capability to achieve rapid and accurate regulation, evident in the swift minimization of transient fluctuations crucial for stability during dynamic conditions. addressing irradiance variations, HYCHOPSO demonstrates its adaptability in regulating the output of the PV array. The system's response to changing environmental conditions underscores its adaptability and responsiveness. Quantitatively, the very short Total Demand Distortion (TDD) calculated according to IEEE519-2014 standardization is 1.1%, well within acceptable limits for a 600 V-bus. The numerical values, including stable active load power at 780 kW, precise frequency stabilization at 50 Hz, and minimal TDD, reinforce HYCHOPSO's efficacy in achieving reliable, stable, and efficient microgrid performance. Overall, the research advocates for the widespread adoption of HYCHOPSO for microgrid control, offering advanced capabilities in power sharing, frequency stabilization, and dynamic response to environmental variations.

### Future work

Subsequent studies may delve into enhancing the HYCHOPSO algorithm, aiming to refine power sharing, frequency stabilization, and dynamic response under various environmental conditions for improved efficiency. Exploring the scalability of HYCHOPSO for larger microgrid systems and its adaptability to diverse grid configurations holds promise for practical applications. Additionally, integrating machine learning techniques into the optimization process and investigating the fusion of HYCHOPSO with advanced control strategies could unlock opportunities for elevating microgrid performance and resilience. Furthermore, studying the challenges and limitation of the HYCHOPSO within real time implementation.

## Data Availability

The authors would like to confirm that all data generated or analysed during this study are included in this published article.

## Abbreviations

A, B, C_2_ and ${r}_{Hat}$: Randomization numbers
$${B}_{i,j}^{t}$$: Turning coefficient
c_1_ and c_2_: Coefficients of optimization parameter which are usually between [0 2] each velocity update
D: Dimension
Eid, Eiq: Error signals in direct current and quadrature current
i: Particle index
I_Bus_600V: Output current on the utility
ILoad: Load current
Iprime_BESS: Current on the primary winding of the transformer connected through the battery energy storage system
Iprime_PV: Current on the primary winding of the transformer connected through PV system
I-PV: PV current
it: Current iteration
Kpdf, Kidf, Kpdv, Kidv, Dfref, Dvref: Drop control parameters
Kpf, and Kif: Control parameter for pi control within active power for maintaining stable frequency with varying power
Kpv, and Kiv: Control parameters for pi control within reactive power for maintaining stable voltage with varying power
lb: Lower boundary
Maxit: Maximum number of iterations.
n: Number of iterations [0, 1,…,n]
Pid, Piq: Control signal of pi controller for direct current and quadrature current error signals
Pout, and Qout: Output active and reactive power
${p}_{n}^{g}$: Global best position
${p}_{n}^{i}$: Local best position
r1, r2: Random number
${\widehat{{\text{r}}}}_{{\text{i}},{\text{j}}}^{-1},{\propto }_{{\text{i}},{\text{j}}}^{{\text{t}}}$: Randomization parameter and step length for cheetah i
RoCoF, RoCoV: Rates of change of frequency and voltage
t: Current hunting time for cheetah
T: Maximum hunting time for cheetah
ub: Upper boundary
${{\text{V}}}_{{\text{n}}}^{{\text{i}}}$: Velocity of particle i at iteration n
Vdconv, Vqconv: Output of the feedforward for direct and quadrature signals
vd_measured,Vq_measured: Output of the feedforward compensation
V-PV, VDC: PV output voltage and voltage on DC bus
W: PSO inertia constant
${{\text{W}}}_{{\text{max}}},{{\text{W}}}_{{\text{min}}}$: Maximum and minimum inertia damping
${X}_{best}^{i},\overline{{X }_{n}^{i}}, {X}_{b}^{i}$: Prey’s position, Cheetah’s current position, and cheetah’s best position
${X}_{i,j}^{t+1},{X}_{i,j}^{t}$: Next and current position of cheetah (i = 1, 2,…,n)
${X}_{n}^{i}$: Position of particle i at iteration n
